# Inactivation of Human Salivary Glutathione Transferase P1-1 by Hypothiocyanite: A Post-Translational Control System in Search of a Role

**DOI:** 10.1371/journal.pone.0112797

**Published:** 2014-11-13

**Authors:** Raffaele Fabrini, Alessio Bocedi, Serena Camerini, Marco Fusetti, Fabrizio Ottaviani, Francesco M. Passali, Davide Topazio, Federica Iavarone, Irene Francia, Massimo Castagnola, Giorgio Ricci

**Affiliations:** 1 Department of Chemical Sciences and Technologies, University of Rome “Tor Vergata”, 00133 Rome, Italy; 2 Department of Cell Biology and Neurosciences, Istituto Superiore di Sanità, 00161 Rome, Italy; 3 Department of Applied Clinical Sciences, University of L’Aquila, 67100 L’Aquila, Italy; 4 Department of Clinical Sciences and Translational Medicine, University of Rome “Tor Vergata”, 00133 Rome, Italy; 5 Institute of Biochemistry and Clinical Biochemistry, Catholic University and/or Institute for Molecular Recognition, National Research Council, 00168 Rome, Italy; University Paris Diderot-Paris 7, France

## Abstract

Glutathione transferases (GSTs) are a superfamily of detoxifying enzymes over-expressed in tumor tissues and tentatively proposed as biomarkers for localizing and monitoring injury of specific tissues. Only scarce and contradictory reports exist about the presence and the level of these enzymes in human saliva. This study shows that GSTP1-1 is the most abundant salivary GST isoenzyme, mainly coming from salivary glands. Surprisingly, its activity is completely obscured by the presence of a strong oxidizing agent in saliva that causes a fast and complete, but reversible, inactivation. Although salivary α-defensins are also able to inhibit the enzyme causing a peculiar half-site inactivation, a number of approaches (mass spectrometry, site directed mutagenesis, chromatographic and spectrophotometric data) indicated that hypothiocyanite is the main salivary inhibitor of GSTP1-1. Cys47 and Cys101, the most reactive sulfhydryls of GSTP1-1, are mainly involved in a redox interaction which leads to the formation of an intra-chain disulfide bridge. A reactivation procedure has been optimized and used to quantify GSTP1-1 in saliva of 30 healthy subjects with results of 42±4 mU/mg-protein. The present study represents a first indication that salivary GSTP1-1 may have a different and hitherto unknown function. In addition it fulfills the basis for future investigations finalized to check the salivary GSTP1-1 as a diagnostic biomarker for diseases.

## Introduction

Saliva is a complex mixture which contains more than 3,000 different proteins many of which may be associated with a disease phenotype and can be very informative for human disease detection [1]. Whole saliva represents an attractive diagnostic fluid because its collection is easy, noninvasive and rapid to obtain without the need for specialized health-care workers and equipments. A large number of medically valuable analytes in saliva have gradually been unveiled and some of them represent potential biomarkers for different diseases including autoimmune, bacterial, cardiovascular and viral diseases and cancer [2].

The use of saliva as a diagnostic fluid was until now limited by circadian variation and polymorphisms, but recent studies [3]–[5] have investigated these aspects, demonstrating that saliva based diagnostics may offer a robust alternative for clinicians to use in the near future to make clinical decisions and predict post treatment outcomes.

In this context, enzymes belonging to the glutathione transferase superfamily are interesting. These enzymes, abundantly expressed in all human tissues, are devoted to cell protection, catalyzing the conjugation of glutathione (GSH) to the electrophilic centre of many toxic and carcinogenic compounds [6]. A number of additional functions of specific GST isoenzymes have been also discovered, including the peroxidase activity [6], an anti-apoptotic role through the binding to cJNK [7], and the protection against nitrosylative stress [8]. GSTs display also non-enzymatic ligandin properties [9]. These enzymes are often overexpressed in tumor tissues and thus considered an important marker of early tumor development [10]. Recently, a direct interaction has been described between the GSTP1-1 and the TRAF domain of TNF receptor-associated factor 2 [11]. In addition, a particular GST isoenzyme present in the erythrocytes, the GSTP1-1, is overexpressed in case of increased blood toxicity as it occurs in nephropathic patients [12] and in healthy subjects living in polluted areas [13]. Thus, erythrocyte GSTP1-1 is considered a biomarker of blood toxicity. Human cytosolic GST isoenzymes are grouped in at least seven different classes named Alpha, Pi, Mu, Theta, Omega, Zeta, and Sigma [14]. While the distribution and expression of these isoenzymes in many human tissues are well known, the presence and the identity of GSTs in saliva is still unclear and only reported by a few authors, with contradictory evidences. In particular, Sreerama and coworkers observed the presence of Alpha, Pi and Mu class GSTs in saliva of healthy subjects only upon ingestion of large amounts of coffee and broccoli [15]. On the contrary, two proteomic studies revealed the presence only of GSTP1-1 [16], [17] while Fang et al. found both GSTP1-1 and GSTA1-1 [18]. In any case, these proteomic studies gave no quantitative estimation of the salivary levels of these enzymes.

The present study proposes to define which specific GST isoenzymes are mainly present in human saliva, their possible interaction with salivary components and to develop a simple method for their quantification. Results are also given for the level of the salivary GST in healthy subjects.

## Materials and Methods

### Materials

1-chloro-2,4-dinitrobenzene (CDNB), GSH, oxidized glutathione (GSSG), cystine, potassium borohydride (KBH_4_), dithiothreitol (DTT), lactoperoxidase (LPO) from bovine milk, hydrogen peroxide (H_2_O_2_), 5,5′-dithiobis(2-nitrobenzoic acid) (DTNB), Bio-gel P2 and all other reagents were purchased from Sigma-Aldrich (St. Louis, USA) and used without further purification. α-defensin 1 and 2 were Peptanova (Sandhausen, Germany) products.

### Ethic Statement

30 healthy volunteers (age ranging from 20 to 65 years; 15 men, 15 women) were recruited. The experiments were undertaken with the understanding and written consent of each subject. The study methodologies conformed to the standards set by the Declaration of Helsinki. The study methodologies were approved by local ethics committee of University of Rome ‘Tor Vergata’.

### Salivary Sample

For three consecutive days, human salivary samples have been collected every day with a minimally-invasive standard procedure using Salivette code blue (Sarstedt AG & Co., Nümbrecht, Germany). Samples were collected three times per day fasting, before brushing teeth in the morning immediately after wakening (8∶00 a.m.), before lunch (11∶00 a.m.), and in the afternoon (3∶00 p.m.). The swab was gently chewed for 1 min and then placed into the Salivette tube. The sample were stored at 4°C and in the same morning centrifuged at 10,000×*g* for 10 min at room temperature. The filtered sample was analyzed for protein content and glutathione transferase activity by following the standard reactivation procedure described below.

### Enzyme activity and protein determination

Each saliva sample was incubated for 45 min at 37°C in a dry block thermostat with 5 mM final concentration of DTT. This protocol represents the optimized procedure for reactivation of oxidized salivary GST. The glutathione transferase activity was assessed by the classical method described by Habig and Jakoby [19], using 1 mM CDNB and 1 mM GSH in 0.1 M potassium phosphate buffer, pH 6.5 (25°C). The specific activity of purified GSTP1-1 at 25°C is 100 U/mg.

The protein content was estimated by Warburg-Christian equation:
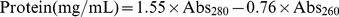
where Abs at 280 nm (proteins) and Abs at 260 nm (nucleic acids) are corrected for scattering (Abs at 500 nm). Moreover, the protein concentration was also randomly estimated using the Lowry method [20] and compared with the Warburg-Christian technique. The difference does not exceed the 7%.

### Glutathione transferase variants

Recombinant GSTP1-1, single variants C47S-GSTP1-1 and C101S-GSTP1-1 and double variant C47S-C101S-GSTP1-1 were expressed in *E. coli* and purified as reported previously [21].

### Chromatography on Bio-gel P2

The chromatography was performed by using a column (30×1.5 cm) packed with a Bio-gel P2 resin. The column was conditioned with ultrapure H_2_O. Molecular markers or authentic saliva were loaded onto the column and eluted by ultrapure H_2_O at 0.6–1.0 mL/min at room temperature. The molecular markers were GSH (MW = 307.3), reduced DTNB (TNB) (MW = 198.2), cysteine (MW = 121.2), phenol (MW = 94.1), SCN^–^ (MW = 58.1).

### Mass spectrometry analysis

High-resolution HPLC-ESI-MS/MS experiments were carried out using an Ultimate 3000 Micro HPLC apparatus (Dionex, Sunnyvale, CA, USA) equipped with a FLM-3000-Flow manager module and coupled to an LTQ Orbitrap XL apparatus (Thermo Fisher Scientific, Waltham, MA). The chromatographic column was a Zorbax 300 SB-C8 (3.5 µm particle diameter; column dimension 1 mm i.d. × 15 cm). High-resolution HPLC-ESI-MS experiments were performed by using the following eluents: (A) 0.1% formic acid in water and (B) 0.1% formic acid in acetonitrile-water 80/20 (v/v). The applied gradient was 0–4 min 5% B, 4–34 min from 5 to 50% B (linear), 34–54 min from 50 to 90% B (linear), at a flow rate of 80 µL/min. High-resolution positive MS/MS spectra were collected in data-dependent acquisition mode; the three most intense multiply charged ions were selected in a time window of 3 milliseconds and fragmented by using collision induced dissociation (35% normalized collision energy) and spectra were recorded. Tuning parameters were: capillary temperature 250°C, source voltage 4 kV, capillary voltage 48 V, tube lens voltage 170 V.

### Preparation of HOSCN/OSCN^–^ and 5-thio-2-nitrobenzoic (TNB) stock solutions

Hypothiocyanite (HOSCN/OSCN^–^) was prepared by the LPO-catalyzed reaction of SCN^–^ with H_2_O_2_ [22]. Stock solution of TNB was generated by reduction of DTNB. About 10 mg of KBH_4_ was added to 2 mL of 1 mM DTNB in 10 mM potassium phosphate buffer pH 7.4 and the solution was mixed for 10 min at room temperature until a constant orange color was formed. The excess of KBH_4_ was destroyed by adding a few drops of HCl (10 M). The solution was neutralized with some drops of NaOH 10 N to reach a final pH of 5–6. TNB concentration was estimated spectrophotometrically measuring the absorbance at 412 nm (ε_412nm_ = 14.1 mM^−1^ cm^−1^). Finally, the concentration of HOSCN/OSCN^–^ stock solutions were measured by detecting the loss of absorbance at 412 nm after adding an excess of TNB. The stoichiometry of the reaction between HOSCN and TNB is 1∶2.

## Results and Discussion

### GST activity in human saliva

**Figure 1:**
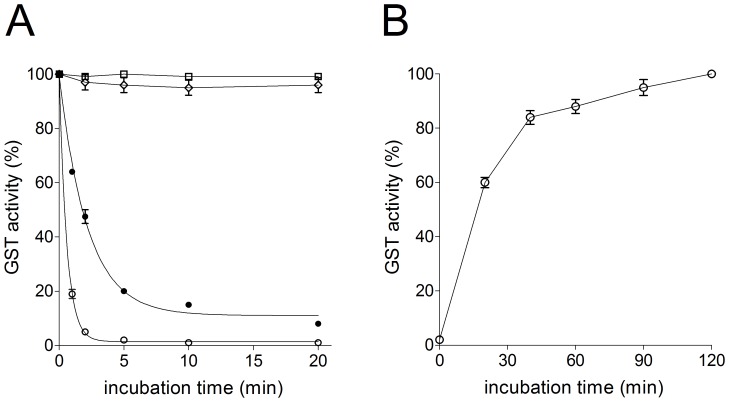
Inactivation of Alpha, Mu and Pi class GSTs by human saliva. (A) GSTP1-1 (open circle) (20 pmoles), GSTA1-1 (open square) (20 pmoles), GSTM2-2 (open diamond) (20 pmoles) were incubated (25°C) with 70 µl of saliva. GSTP1-1 was also incubated with the same salivary sample diluted 1∶10 (full circle). (B) GSTP1-1 (open circle) inactivated as in A for 20 min and then treated with 1 mM DTT at 37°C. Each experiment was performed in triplicate (i.e. three different spectrophotometric determinations on the same salivary sample). Error bars represent SEM.

**Figure 2:**
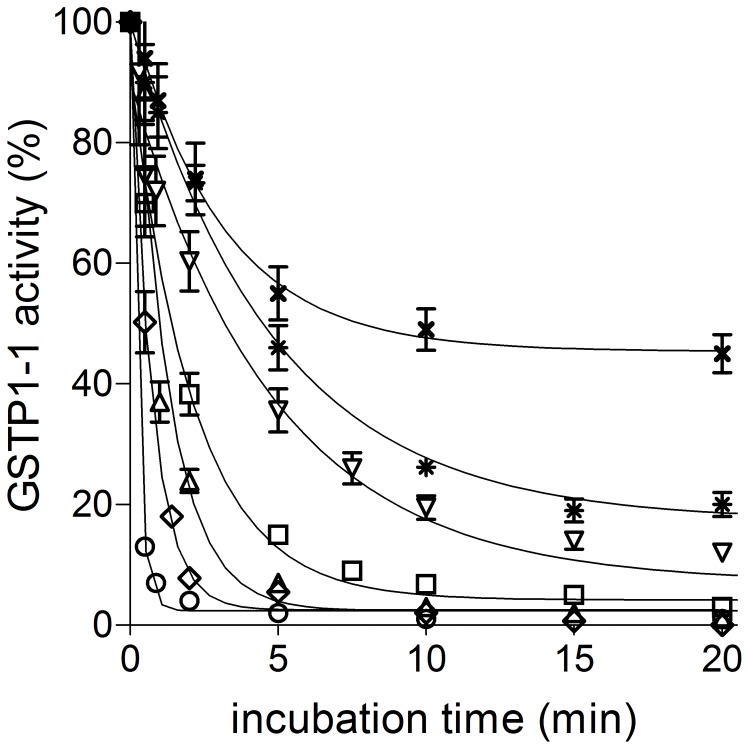
Stoichiometry of GSTP1-1 inactivation by saliva. GSP1-1 (20 pmoles) was incubated with human saliva (70 µl) as such or at variable dilutions (*open circle*: no dilution; *open diamond* 1∶5; *open triangle*: 1∶10; *open square* 1∶20; *reverse triangle* 1∶40, *asterisk* 1∶50 and *cross* 1∶100) and at different incubation time (25°C). Each experiment was performed in triplicate (i.e. three different spectrophotometric determinations on the same salivary sample). Error bars represent SEM.

Under the usual assay conditions for GST activity (1 mM GSH and 1 mM CDNB in 0.1 M potassium-phosphate buffer, pH 6.5) no detectable catalysis could be observed in 20 different saliva samples obtained from healthy subjects as described under Experimental Procedures. To check the possible presence of salivary GST inhibitors, we performed various controls. Saliva samples were supplemented with variable amounts of GSTP1-1, GSTM2-2 or GSTA1-1, as representative GSTs belonging to the most abundant classes expressed in humans (Alpha, Pi and Mu) [6]. While the activity of GSTA1-1 and GSTM2-2 in the samples was satisfactory (95–100%), the one of GSTP1-1 was less than 1% (Figure 1A). The observed inactivation was reversible and likely due to an oxidative event. In fact, treatment of these samples with 1 mM DTT at room temperature for 120 min restored the expected activity (Figure 1B). Really, exogenous GSTP1-1 underwent a fast inactivation in saliva (Figure 1A) and even diluted saliva samples were still able to inactivate this enzyme in a few minutes (Figure 2). Saliva samples not supplemented with exogenous GSTP1-1, and treated with DTT, also disclosed a detectable GST activity ranging from 30 to 50 mU per mg of salivary proteins. The observed fast inactivation is not a catalytic event but likely caused by a stoichiometric interaction of the enzyme with a component of the saliva. In fact, fixed amounts of saliva incubated with variable over-stoichiometric levels of GSTP1-1 gave the same amount of inhibited GSTP1-1 (not shown). Furthermore, incubation of a fixed amount of the enzyme with saliva samples at increasing dilutions gave different levels of inhibited enzyme correlated to the different dilutions (Figure 2). From these data and assuming a 1∶1 stoichiometry for the reaction GST-inhibitor, we estimated the concentration of the inhibitor from 5 to 15 µM in ten different saliva samples.

### Mass spectrometry

The GSTP1-1 wild-type is an homodimeric enzyme with four cysteines (14, 47, 101, and 169) in each subunit (Figure S1). Mass spectrometry demonstrated that in the native enzyme the inactivation occurring in saliva is due to the formation of an intra-chain disulfide bridge. In fact, the molecular mass of the inactivated GST displays a value corresponding to a single monomer but with two Da units lower than the native subunit (Table 1) (Figure S2). This result excludes that this disulfide may involve cysteines of the adjacent subunit as well as mixed disulfide of the protein with small salivary proteins or with small thiols like GSH or Cys. Cys47 and Cys101 are most likely involved because, as observed in the past, it is mainly this disulfide that is formed under oxidizing conditions [23]–[25]. However, in the absence of Cys101, alternative disulfides can be also formed, as indicated by the loss of two mass units found using the Cys101Ser variant. MS/MS fragmentation of the intact proteins was very poor (due to the disulfide bridge). Only few fragments of the ***y*** and ***b*** series around 133–150 residues were detected, suggesting that Cys169 is not involved in the formation of these alternative disulfides (data not shown).

**Table 1:** Mass for GSTP1-1 and C101S variant after reaction with human saliva.^a.^

	[M+H]^1+^ *(m/z)*	[M+H]^1+^ *(m/z)*	ΔM
GSTP-1 (Swiss Prot code P09211)	23340.092	23342.030	1.938
GSTP-1 (Met_1_ missing)	23209.040	23210.989	1.949
GSTP-1 C101S mut.	23324.155	23326.053	1.898
GSTP-1 C101S mut. (Met_1_ missing)	23193.117	23195.012	1.895

a GSTP1-1 and C101SGSTP1-1 were incubated with human saliva (30 min at 25°C) (mass data are reported on the left column). On the central column, the theoretical values for the reduced GSTP1-1 and C101SGSTP1-1 are also reported.

### Kinetics experiments with GSTP1-1 variants

**Figure 3:**
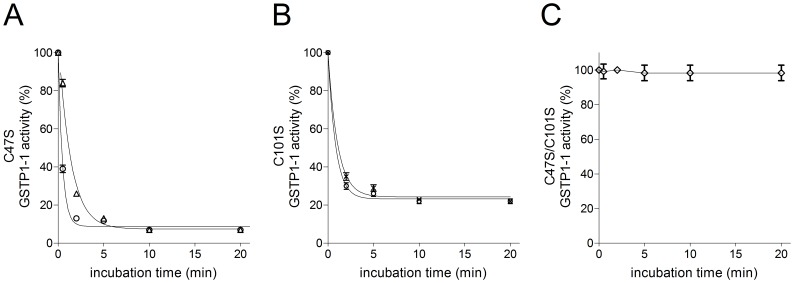
Inhibition of cysteine variants of GSTP1-1 by saliva. (A) C47S variant (20 pmoles) incubated at 25°C with 70 µl of saliva (open circle) or saliva 1∶20 diluted (open triangle). (B) C101S variant (20 pmoles) incubated with 70 µl saliva (open circle) or saliva diluted 1∶10 (cross). (C) C47S/C101S double variant (20 pmoles) incubated with 70 µl of saliva (open diamond). Each experiment was performed in triplicate (i.e. three different spectrophotometric determinations on the same sample). Error bars represent SEM.

Experiments with specific Cys variants of GSTP1-1 further confirmed the involvement of Cys47 and Cys101 in the observed inactivation. As shown in Figure 3, replacement of both these residues by Ser fully prevented the inactivation due to the salivary inhibitor. Conversely, single point mutations involving only Cys47 or Cys101 allowed salivary inactivation but to a lower extent and with slower kinetics than that observed with native GSTP1-1. The fast and almost complete inactivation observed within the native enzyme confirms that its inactivation mainly involves the oxidation of only Cys47 and Cys101 (Figure 3). As above indicated, alternative intramolecular disulfides can be formed, but only in the absence of Cys47 or Cys101 and possibly involving Cys14.

### GSH and cysteine protect GSTP1-1 from inactivation

The presence of GSH in the saliva samples was found to protect the exogenous GSTP1-1 from inactivation. As Cys47 and Cys101 are located near the G-site, this protection has been first explained on the basis of a steric hindrance of these two cysteines by the bound GSH, as it has been observed using glutathione analogs in the presence of other GSTP1-1 inhibitors [26]. However, the protective effect is still present when GSH is only 10–20 µM, concentrations unable to saturate the G-site (*K_D_* = 100 µM) [27]. Furthermore, also cysteine and other thiols at similar low concentrations display identical behavior suggesting that the protection is due to a direct and stoichiometric interaction of the sulfhydryl group of these compounds with the unknown inhibitor. Interestingly, the minimal amount of GSH able to protect GSTP1-1 (10 µM) is close to the inhibitor level calculated on the basis of the enzyme inhibition.

### Salivary proteins or low molecular mass disulfides as possible agents for GSTP1-1 oxidation

**Figure 4:**
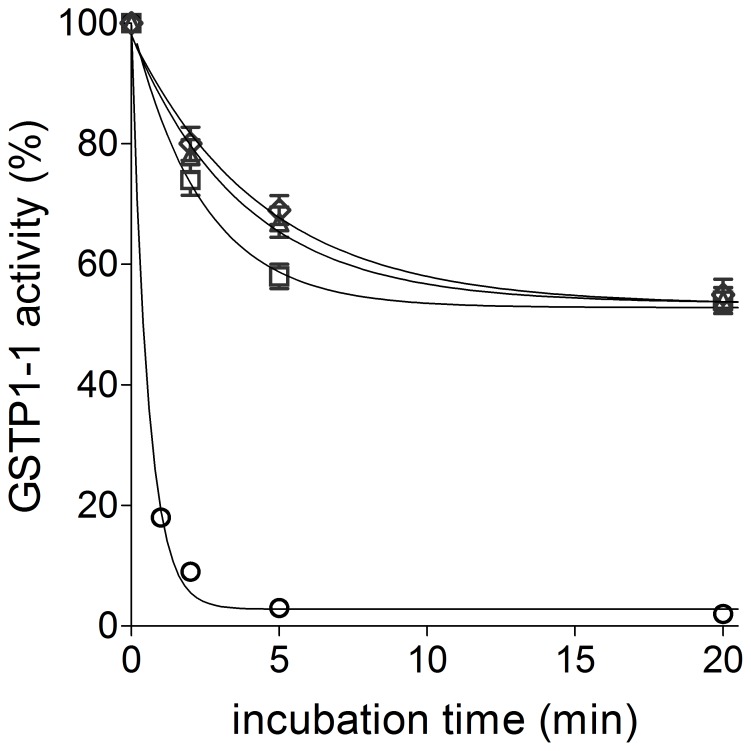
Inactivation of GSTP1-1 by human defensins (HNP-1 and HNP-2). GSTP1-1 (90 pmoles) was incubated at 25°C with HNP-1 (0.54 nmoles) (open triangle), HNP-2 (open diamond) (0.54 nmoles) and both HNP-1 0.27 nmoles and HNP-2 (0.27 nmoles) (open square) in 70 µl (final volume) of potassium phosphate buffer, pH 7.0 (these defensin levels reproduce the average concentration of these proteins in saliva). An identical amount of GSTP1-1 was also incubated with 70 µl saliva (open circle). Each experiment was performed in triplicate (i.e. three different spectrophotometric determinations on the same sample). Error bars represent SEM.

The oxidized GSTP1-1 might be formed through interaction of the native enzyme with some salivary disulfides as shown in Scheme S1. Heat treatment of salivary samples (100°C for 5 min) abolishes the inhibition activity found in saliva (not shown), thus the possible involvement of some salivary protein with reactive disulfides has been considered. Defensins, a group of small antimicrobial proteins present in saliva (about 3.5 kDa for α-defensin 1, 2 and 3) display three disulfide bridges and therefore are possible candidates to interact with the reactive cysteines of GSTP1-1 [28]. In addition their concentration in saliva (5–8 µM) [28] is close to that estimated for the unknown inhibitor. However, incubation of GSTP1-1 with a large excess of α-defensin 1 and/or 2, chosen as representative types of these small proteins, only cause a slow inhibition (Figure 4). In addition, the inhibition does not exceed 50% of the original activity, indicating an half-site inhibition process. This particular behavior has been observed previously for some inhibitors of GSTP1-1 and it probably represents a cooperative self-preservation mechanism finalized to save 50% of the original activity in case of inhibitor attack [29]. Thus the inhibition pattern due to α-defensins is very different from that seen in the presence of authentic saliva, which likely indicates that other saliva components inactivate GSTP1-1 (Figure 4).

A second protein suspect for GSTP1-1 inhibition could be a member of the cystatine family. These proteins do not display any intramolecular disulfide but only one Cys residue located at the end of the polypeptide chain. Recent findings showed that this residue is always present in saliva as mixed disulfide with glutathione or cysteine [30], and it is therefore possible candidate for thiol-disulfide interaction with GSTP1-1. However, also these proteins (molecular mass 11–12 kDa) can be discarded because filtration of human saliva on Amicon Ultra 10K membrane (exclusion size >10 kDa) did not affect the inhibiting activity (data not shown).

**Figure 5:**
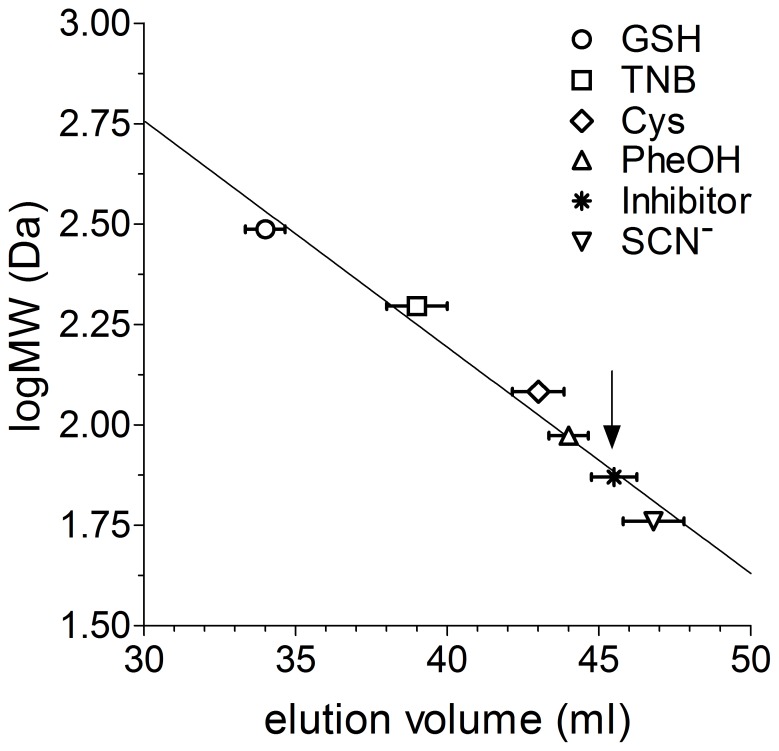
Size-exclusion chromatography of the unknown inhibitor. Experiments were made using a Biogel P-2 resin as described under Experimental Procedures. Molecular markers: GSH, TNB, cysteine, phenol, and thyocianate. The arrow indicates the elution volume of the unknown inhibitor, corresponding to a molecular mass of about 70–85 Da. Error bars represent SEM.

An approximate estimation of the mass of the unknown inhibitor has been obtained by a Biogel P2 chromatography. The inhibitor displays a very low molecular mass, ranging between 70 and 85 Da (Figure 5).

**Figure 6:**
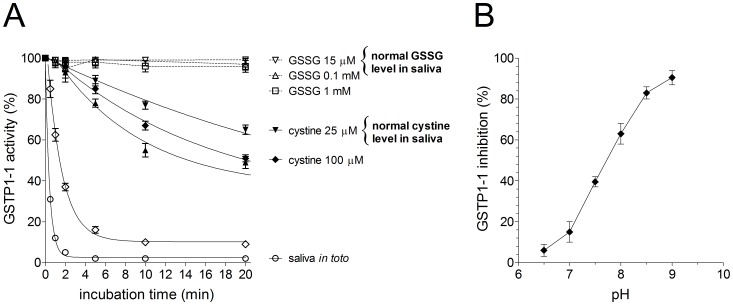
Inactivation of GSTP1-1 by GSSG and CysSSCys. (A) GSTP1-1 (20 pmoles) incubated at 25°C with variable amounts of oxidized glutathione or oxidized cysteine in 70 µl of 0.1 M potassium phosphate buffer, pH 7.0. For comparison the same amount of GSTP1-1 was incubated with 70 µl saliva. (B) pH dependence of the inactivation by cystine (100 µM). The activity was evaluated after 5 min of incubation. Each experiment was performed in triplicate (i.e. three different spectrophotometric determinations on the same sample). Error bars represent SEM.

GSSG and cystine are endogenous disulfides with low mass and thus possible candidates; their average concentrations in saliva are 16 µM and 26 µM, respectively, just in the estimated range of the inhibitor [31]. However, when incubated *in vitro* with authentic GSTP1-1 these disulfides only cause very slow rate of inactivation even at concentrations very much higher than those found *in vivo* (Figure 6A). In addition, the pH dependence of this reaction (Figure 6B) is that expected for a simple thiol-disulfide exchange, which usually becomes faster by increasing the pH values. On the contrary, the pH dependence of the inactivation rate due to saliva is peculiar showing a decrease above pH 7.0 (see below).

### Hypothiocyanite is the real GSTP1-1 inhibitor

**Figure 7:**
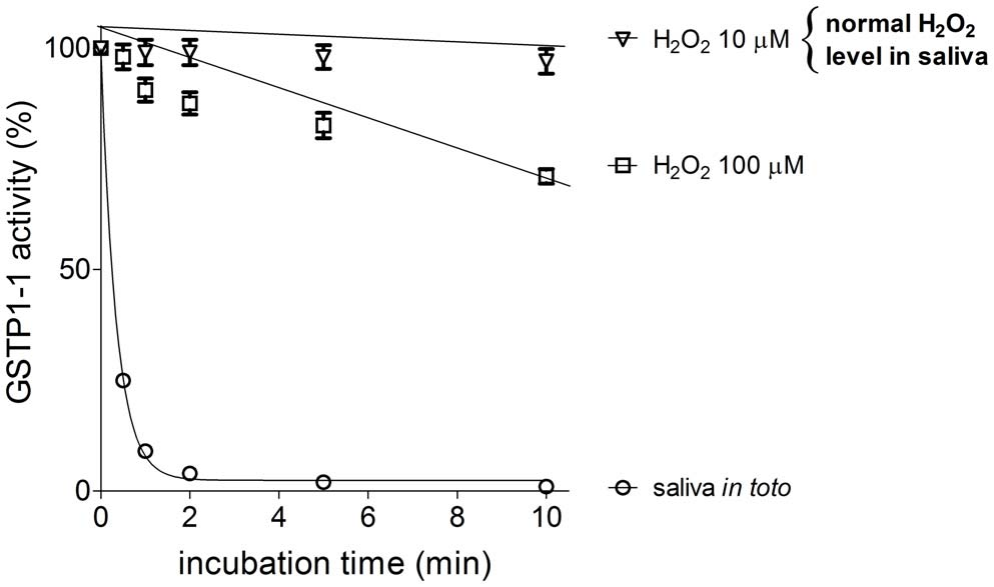
Inhibition of purified GSTP1-1 by H_2_O_2_. GSTP1-1 (20 pmoles) was incubated at 25°C with two different concentrations of H_2_O_2_, 10 µM, corresponding to the physiological level (open triangle) and 100 µM (open square) in 70 µl potassium phosphate buffer, pH 7.0. For comparison GSTP1-1 (20 pmoles) was also incubated with 70 µl of saliva (open circle). Each experiment was performed in triplicate (i.e. three different spectrophotometric determinations on the same sample). Error bars represent SEM.

A few low mass oxidizing compounds lacking disulfides are also present in saliva. Among them H_2_O_2_ has been first considered. H_2_O_2_ is actively produced in saliva by bacteria but mainly by epithelial Nox/Duax NAD(P)H oxidases [32] and it is able to oxidize GSTP1-1 [33]. Interestingly, its estimated average concentration in human saliva (10 µM) [34] is close to that calculated for the unknown inhibitor. However, this concentration and even much higher levels (100–200 µM) only cause very slow oxidative inactivation of GSTP1-1, not comparable with the very fast inhibition observed with authentic saliva (Figure 7). Furthermore, pre-incubation of human saliva with catalase did not decrease or abolish the inhibition event.

**Figure 8:**
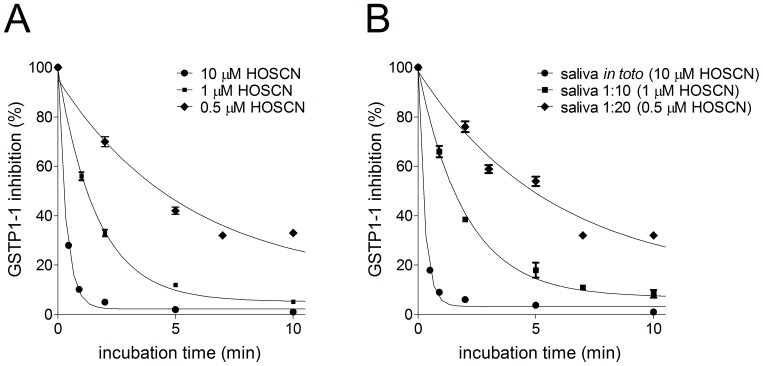
Inhibition pattern of GSTP1-1 by salivary samples or authentic HOSCN. (A) Purified GSTP1-1 (20 pmoles) incubated with authentic HOSCN (10, 1, and 0.5 µM). (B) Purified GSTP1-1 (20 pmoles) was at 25°C incubated with 70 µl of saliva (full circle) (estimated inhibitor concentration 10 µM) or at different dilutions (full square 1∶10; full diamond 1∶20). Each experiment was performed in triplicate (i.e. three different spectrophotometric determinations on the same sample). Error bars represent SEM.

**Figure 9:**
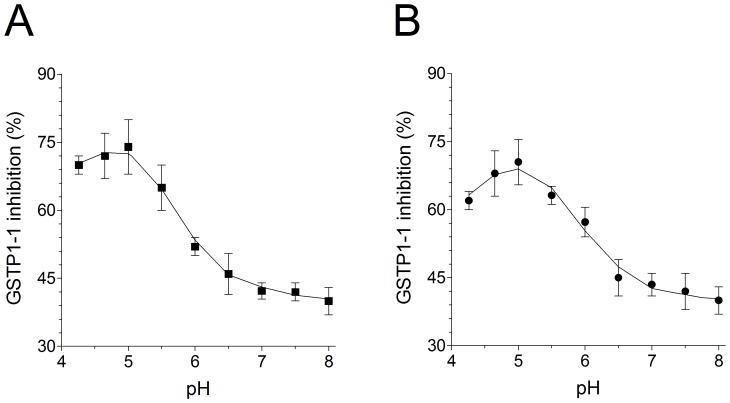
pH dependence of GSTP1-1 inhibition. (A) Purified GSTP1-1 (20 pmoles) incubated with saliva (estimated inhibitor concentration 5 µM) at different pH values (from 4.3 to 8.0) with suitable additions of 0.1 M potassium dihydrogen phosphate or 0.1 M potassium monohydrogen phosphate. After incubation (1 min, 25°C) the activity was measured. (B) GSTP1-1 incubated as in (A) with authentic HOSCN 5 µM. Each experiment was performed in triplicate (i.e. three different spectrophotometric determinations on the same salivary sample). Error bars represent SEM.

**Figure 10:**
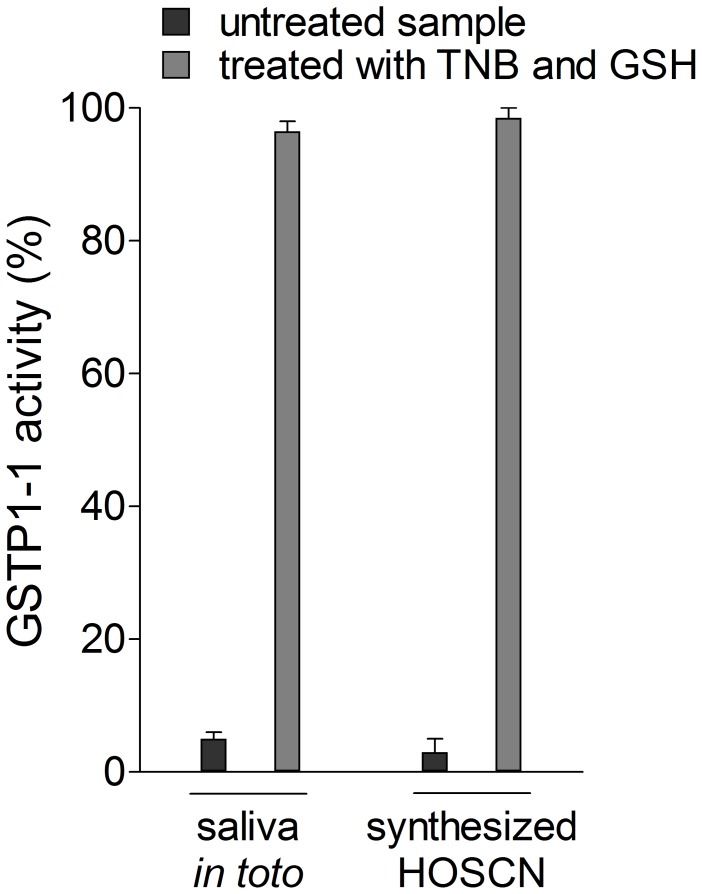
Interaction of GSTP1-1 with saliva or authentic OSCN^−^ after reaction with TNB. GSTP1-1 (20 pmoles) was incubated for 10 min at 25°C in 70 µl of saliva (estimated level of OSCN**^−^** = 5–10 µM). In a second experiment, before the addition of GSTP1-1, the saliva sample was treated with 10 µM of TNB, and then with the same amount of GSH to remove DTNB. The same experiment was also performed in the absence of saliva, using authentic hypothiocyanite (5 µM) in potassium phosphate buffer pH 7.0. Each experiment was performed in triplicate (i.e. three different spectrophotometric determinations on the same sample). Error bars represent SEM.

Hypothiocyanite (OSCN^−^) is another oxidizing salivary component that displays a molecular mass compatible to that predicted by the exclusion chromatography on Biogel P2 (molecular mass = 74.1 Da). This compound represents one of the most active salivary antimicrobial agent formed in virtue of large amounts of salivary thiocyanate, H_2_O_2_ and salivary lactoperoxidase. Hypothiocyanite is not stable but its level in saliva is stabilized at about 10–20 µM concentration by the presence of a large excess of thiocyanate and a continuous generation of H_2_O_2_ by salivary bacteria and by epithelial Nox/Duox family NADPH oxidases [32], [35], [36]. A first indication that this compound may be the real inhibitor for GSTP1-1 has been obtained by using authentic OSCN^−^. Kinetics and the peculiar pH dependence of GSTP1-1 inactivation (Figure 8 and 9) are almost identical to those found using salivary samples. Moreover, using TNB as specific OSCN^−^ titrant [22], we found that the concentration of this compound in saliva is almost identical to that calculated on the basis of GSTP1-1 inhibition (not shown). Furthermore, after titration of salivary OSCN^−^ with stoichiometric amount of TNB and subsequent removal of DTNB with GSH (DTNB is a strong inhibitor of GSTP1-1 [23]), the salivary sample becomes unable to inactivate GSTP1-1 (Figure 10). A more accurate analysis allowed us to calculate the second order kinetic constants for the inactivation of GSTP1-1 in the presence of OSCN^−^ or H_2_O_2_ (*k*_OSCN¯_ = 2.5×10^5^ M^−1 ^min^−1^; *k*_H2O2_ = 3×10^2 ^M^−1 ^min^−1^). The ratio *k*_OSCN¯/_ *k*_H2O2_ is about 800. In other words, hypothiocyanite is about 800 times more efficient than H_2_O_2_ in the GSTP1-1 inactivation.

### Reactivation of GSTP1-1

**Figure 11:**
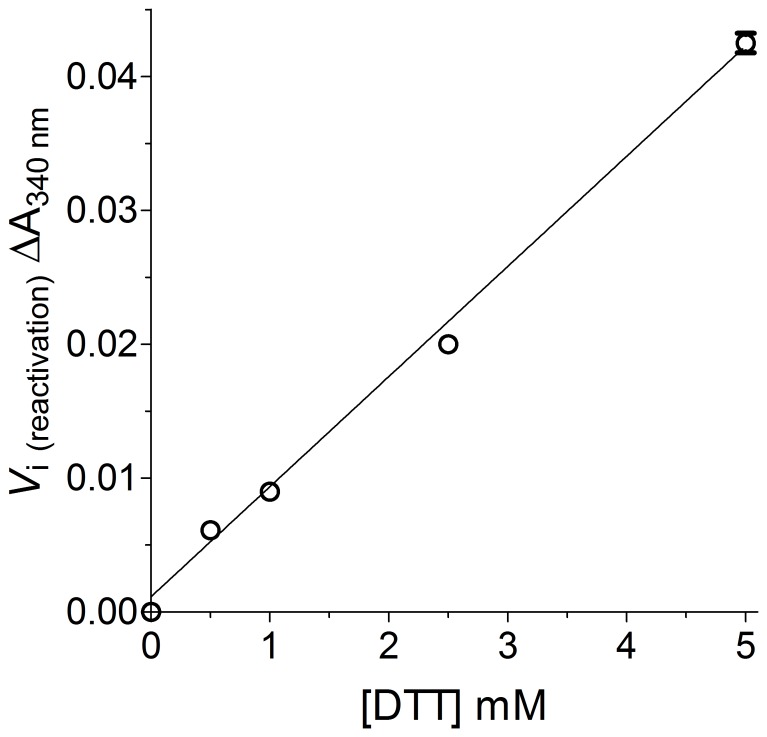
Reactivation rate of GSTP1-1. Purified GSTP1-1 (20 pmoles) was incubated with HOSCN (10 µM, final concentration) for 20 min, 25°C. Then the samples were incubated with DTT at different concentrations for 2.5 min, 37°C in potassium phosphate buffer 0.1 M, pH 8.4, and the activity was measured. Each experiment was performed in triplicate (i.e. three different spectrophotometric determinations on the same sample). Error bars represent SEM.

The oxidized form of GSTP1-1 is characterized by a considerable structural re-arrangement as suggested by the change in electrophoretic pattern and by the modification of the CD and fluorometric spectra [23]. Cys47 is located 1.9 nm away from Cys101 and even further from Cys14 and Cys169. Similar large distances separate Cys101 from Cys169 and Cys14. It is therefore reasonable that significant structural changes follow an intra-chain disulfide formation [37]. Reactivation experiments permit us to examine the process and also obtain insights in the molecular mechanism. Kinetics of reactivation by DTT depends linearly on DTT concentration (Figure 11). This suggests that in our conditions the rate limiting step is the chemical reduction of the protein disulfide and not the structural rearrangement that likely follows the chemical step. In the latter case the reactivation should be almost independent on the DTT concentration. Kinetics of reactivation shows a pH dependence as that expected for a simple thiol-disulfide exchange, with a progressive increase of the rate at higher pH values. The optimized conditions for a fast and quantitative reactivation of the salivary GSTP1-1 were 5 mM DTT in 0.1 M potassium-phosphate buffer, pH 8.0, 37°C for 45 min.

### Salivary GSTP1-1 in healthy subjects

**Figure 12:**
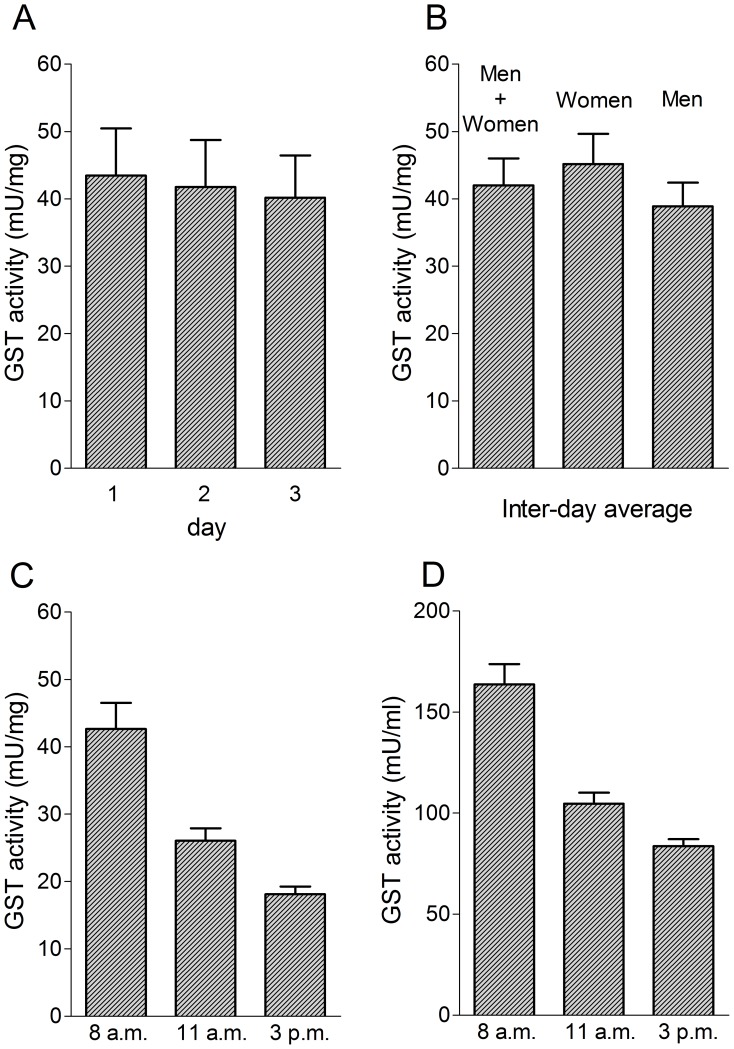
Inter-day and intra-day levels of salivary GSTP1-1. (A) Inter-day activity of GSTP1-1 (early morning recovery -Men+women). (B) Sex distribution of the salivary GSTP1-1 as average of three days (early morning recovery). (C) Average of salivary GSTP1-1 (men+women) during three days at different time of saliva recovery (8 am, 11 am, 3 pm). Values expressed as mU/mg of salivery proteins. (D) as in (C) with GSTP1-1 activity expressed as mU/ml of saliva. Error bars represent SEM.

The salivary GSTP1-1 activity of thirty healthy, non-smoking subjects (15 women and 15 men) has been measured in three consecutive days using the reduction procedure mentioned above. Saliva samples were collected as described under “Experimental procedures” in the early morning at least one hour from consumption of water or food. The total average of 90 recoveries (three for each subject in different days) was 42±4 mU/mg (Figure 12A). The inter-day variation was lower than 8%. Women display about 15% higher values than men (Figure 12B). No correlation has been found between GST activity and age (*r* ^2^ = 0.07 *p* = 0.18). Interestingly, salivary GSTP1-1 concentration (mU/mg) follows a circadian rhythm with the lowest level in the afternoon (Figure 12C). A similar trend has been found for the GSTP1-1 expressed as mU/ml (Figure 12D).

Finally, we noted that no irreversible inactivation possibly due to proteolytic break-down occurs in the temporal span from the sample collection to the enzyme analysis (about 1–3 hours at 4°C). In fact an unchanged GST activity was observed if the DTT treatment and the GST activity is made within five min from collection and similar unchanged activity was also found five hours from collection at 4°C. The absence of a detectable turnover allows an approximate estimation of the daily production of the salivary GST i.e. 120 mU/ml. As the total volume of saliva produced by humans is 1 l/day, they produce 120 U of GSTP1-1 per day (at 25°C) corresponding to 1.2 mg of enzyme (the specific activity of purified GSTP1-1 is about 100 U/mg).

## Conclusions

This paper demonstrates that GSTP1-1 is present in saliva and that its activity may be easily measured after a simple reduction step with DTT. Other GST isoenzymes are not represented or are below the limit of detection. This conclusion represents a clarification of the contradictory evidence reported previously in this context [7], [8], [10], [12]. Even if other GSTs may be present in trace amounts in saliva, GSTP1-1 clearly represents the most abundant isoenzyme. Mass spectrometry and site-directed mutagenesis show that in saliva this enzyme is always in an oxidized disulfide form mainly involving Cys47 and Cys101, the most reactive cysteines among the four present in each monomer. The presence of glutathionylated GSTP1-1, which is another possible oxidized form of this enzyme, can be excluded due to the absence of any glutathionylated fragment. The results presented here, such as the particular pH dependence of the rate of inactivation, the diagnostic reaction with TNB and the size exclusion chromatography, clearly demonstrate that hypothiocyanite is the main inactivating compound. Its interaction with GSTP1-1 follows a 1∶1 stoichiometry. Interestingly, OSCN^−^ is more than 800 times more active than H_2_O_2_ in its interaction with GSTP1-1. Hypothiocyanite is a well known thiol-specific reagent naturally occurring in human saliva. Its antibacteric and pro-apoptotic properties arise probably from specific targeting of critical thiol residues. Even if it has been reported that in red blood cells, exposed to 0.1 mM OSCN^−^, a few enzymes, *i.e*. glyceraldehyde dehydrogenase and also glutathione transferase are oxidatively inactivated [38], the present study for the first time shows that hypothiocyanite interaction with GSTP1-1 really occurs in saliva under physiological conditions, and that this reaction explains the apparent absence of this enzyme in saliva. At present, the exact sequence of the chemical events leading to the transferase inactivation can only be speculated. As shown in Scheme S2, two different pathways are possible; one involves the intermediate formation of a sulphenic group of a reactive cysteine and the other the involvement of a thiocyano-derivative, both yielding a final intra-chain disulfide.

By using a simple reactivation procedure, the salivary GST level of 30 healthy subjects has been evaluated during three days, at three different times (Figure 12). Women show a bit higher salivary GSTP1-1 level then men (15%). While the inter-day variation is low (about 8%), the intra-day variation is relevant with the maximal concentration in the early morning i.e. 42±4 mU/mg or 164±10 mU/ml. This value corresponds to about 70 nM of GSTP1-1 in the total saliva. The presence of hundred times higher concentrations of OSCN^−^ (10–20 µM) in saliva explains the total absence of transferase activity in all tested salivary samples. These data represent the first reference values for future investigations finalized to check the salivary GST as biomarker for specific diseases. Stability during storage, and changes of levels caused by specific diseases will be object of a subsequent investigations. What is the origin of the salivary GSTP1-1? Does it come from oral epithelial cell lysis due to natural or mechanical stress or, alternatively is it a salivary gland secretion? The second suggestion is more probable. In fact, the intra-day variation of salivary GSTP1-1 (see Figure 12) agrees with the well know circadian rhythm of gland secretion and is less compatible with an epithelial cell lysis. Furthermore, the relevant amount of salivary GSTP1-1 produced every day (see below) is improbable to come from an oral cell exfoliation.

The presence of an inactive oxidized form of GSTP1-1 in saliva is a surprising finding and represents an interesting enigma. In previous studies we and other researchers demonstrated that, except for Cys47, replacement of Cys101 and all other cysteines by Ser perturbs the enzymatic activity of this enzyme only slightly or not at all [21], [39]. It is thus somewhat surprising that all the four cysteines found in the human GSTP1-1 are conserved exactly in the same sequence location in many mammalian GSTP1-1 such as cow, pig, horse etc. This strict conservation during evolution seems paradoxical because these cysteines represent a sort of Achilles’ heel for all these transferases because they may be easily inactivated by oxidation. However, nothing in nature is casual, and it is possible that the oxidized GSTP1-1 may have some physiological relevance. One possibility is that it could display a peculiar catalytic or ligandin activity yet to be discovered, or alternatively it could act as signal for cellular receptors. The suggestion for a biological role is supported by the relevant energetic cost of GSTP1-1 synthesis in saliva. As above calculated, humans produce 1.2 mg of salivary GSTP1-1 every day i.e. about 108 mg during three months. This value should be compared to the amount of GSTP1-1 found in human blood (5.6 U/g_Hb_ at 37°C [12] i.e. 4700 U in 6 l of blood corresponding to 3380 U at 25°C, i.e. about 34 mg of protein. This value represents a steady-state level but during the erythrocyte life, the degradation, if it occurs, is negligible. Preliminary data indicate an unchanged GST activity during weeks (blood at 37°C supplemented by glucose) and previous studies demonstrated a strict correlation between GST activity and GST protein expression [40]. Thus, we may assume that about 34 mg of GST represents the total amount produced during three months (the erythrocyte mean life) indicating that the energetic cost for the salivary GSTP1-1 synthesis is three times higher than the blood GSTP1-1. In conclusion, the salivary GSTP1-1 cannot be considered a noise expression, but its presence suggests an hidden function to be discovered probably independent of its classical conjugating activity. Work is in progress to clarify this question which could open new perspectives for one of the most studied enzymes.

## Supporting Information

Figure S1 **Tridimensional structure of human GSP1-1.** The structure of the dimeric enzyme (chain A and B) (PDB id: 6GSS) is shown in ribbon while the cysteine residues are reported in ball-and-stick (yellow). The picture was drawn using UCSF Chimera. [Reference: Pettersen EF, Goddard TD, Huang CC, Couch GS, Greenblatt DM, et al. (2004) UCSF Chimera. A visualization system for exploratory research and analysis. J Comput Chem 13∶1605–1612.]
(TIF)

Figure S2 **Mass spectrometry of wild type GSTP1-1 and the C101S variant after reaction with saliva.** (A) Total Ion Current chromatogram of human GSTP1-1. (B) ESI-IT spectrum of GSTP1-1. (C) deconvoluted ESI spectrum of GSTP1-1. (D) Total Ion Current chromatogram of C101S variant. (E) ESI-IT spectrum of GSTP1-1. (F) deconvoluted ESI spectrum of C101S variant.
(TIFF)

Scheme S1 **Hypothetical GSTP1-1 oxidation by a disulfide inhibitor.**
(TIF)

Scheme S2 **Alternative inactivation pathways by hypothiocyanite.**
(TIF)
