# Supplementary figures and images for: Inactivation of Human Salivary Glutathione Transferase P1-1 by Hypothiocyanite: A Post-Translational Control System in Search of a Role
Source: PLoS One. 2014 Nov 13;9(11):e112797. doi: 10.1371/journal.pone.0112797 (PMC4231102; doi:10.1371/journal.pone.0112797)

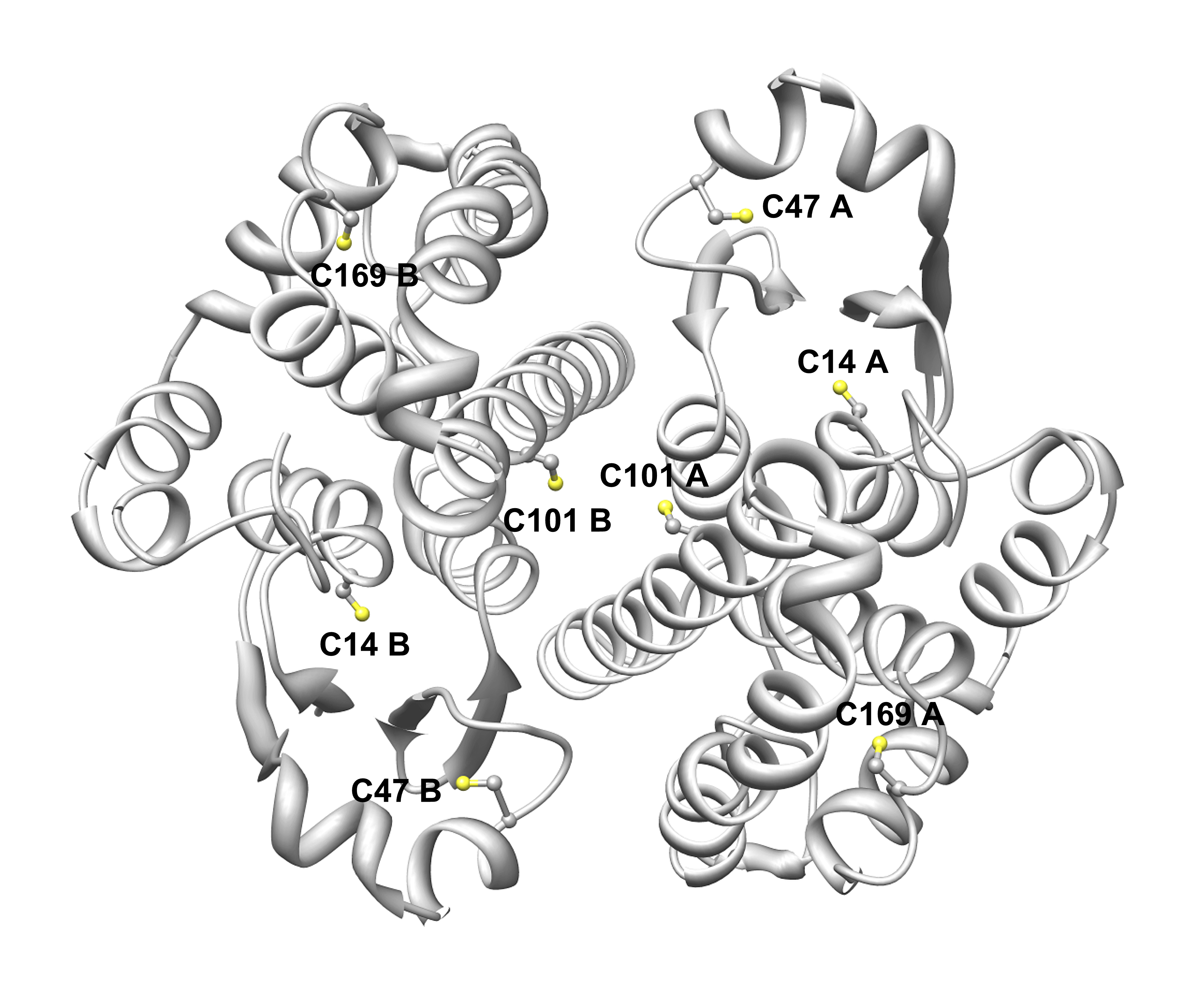

Supplement: Figure S1 — Tridimensional structure of human GSP1-1. The structure of the dimeric enzyme (chain A and B) (PDB id: 6GSS) is shown in ribbon while the cysteine residues are reported in ball-and-stick (yellow). The picture was drawn using UCSF Chimera. [Reference: Pettersen EF, Goddard TD, Huang CC, Couch GS, Greenblatt DM, et al. (2004) UCSF Chimera. A visualization system for exploratory research and analysis. J Comput Chem 13∶1605–1612.] (TIF) [file pone.0112797.s001.tif]

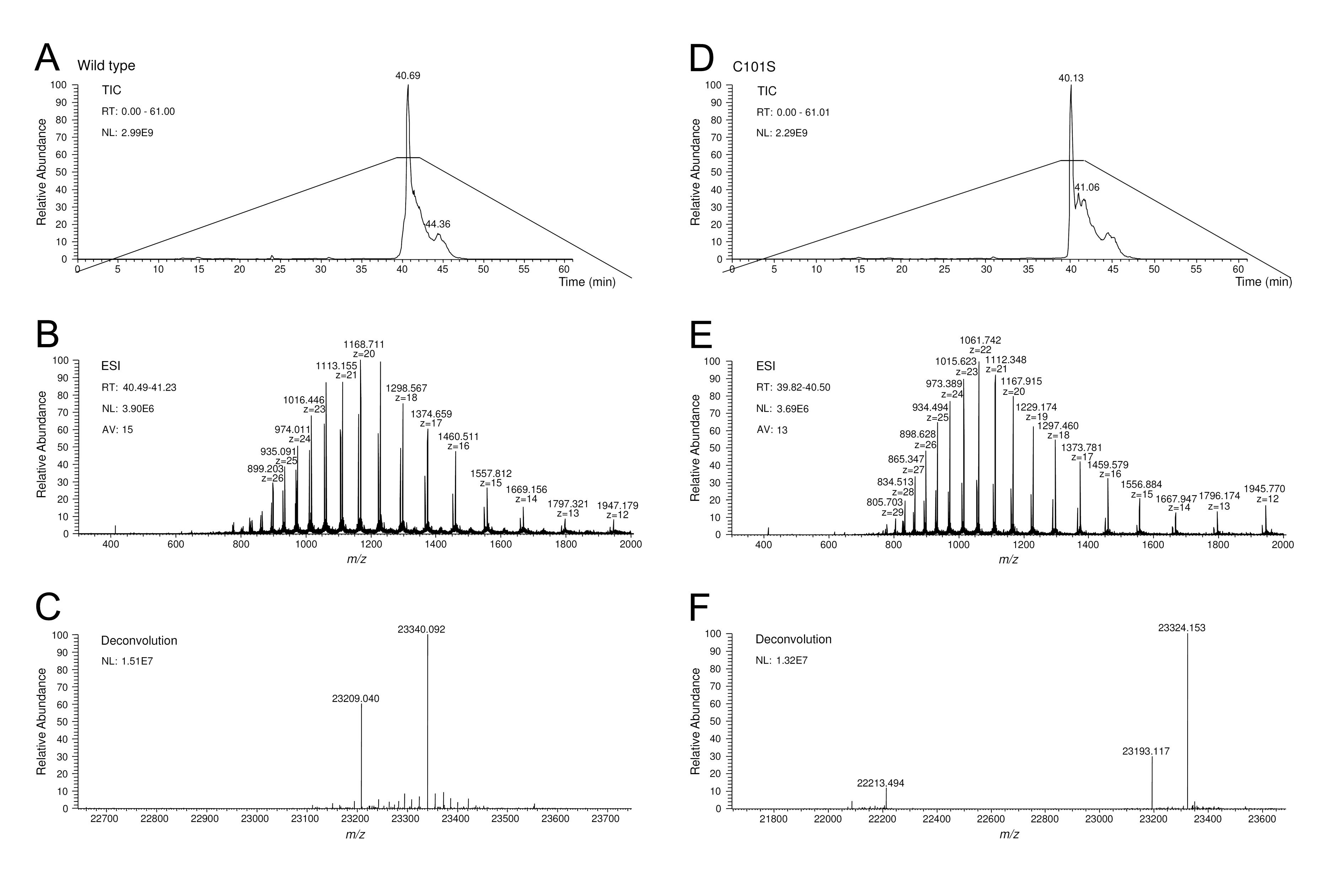

Supplement: Figure S2 — Mass spectrometry of wild type GSTP1-1 and the C101S variant after reaction with saliva. (A) Total Ion Current chromatogram of human GSTP1-1. (B) ESI-IT spectrum of GSTP1-1. (C) deconvoluted ESI spectrum of GSTP1-1. (D) Total Ion Current chromatogram of C101S variant. (E) ESI-IT spectrum of GSTP1-1. (F) deconvoluted ESI spectrum of C101S variant. (TIFF) [file pone.0112797.s002.tiff]
